# Blood pressure variability and cognitive dysfunction: What is the cause and what is the consequence?

**DOI:** 10.1111/jch.14370

**Published:** 2021-10-26

**Authors:** Luis Giménez‐Miranda, Luis M. Beltrán‐Romero, Pablo Stiefel

**Affiliations:** ^1^ Unidad Clínica de Atención Médica Integral (UCAMI) y Laboratorio de Epidemiología Clínica y Riesgo Vascular, Instituto de Biomedicina de Sevilla (IBiS) Hospital Universitario Virgen del Rocío/CSIC/Universidad de Sevilla Seville Spain

Dear Sir,

We read with great interest the paper entitled “Blood pressure variability and cognitive dysfunction: A systematic review and meta‐analysis of longitudinal cohort studies” recently published in the *Journal of Clinical Hypertension*.^1^ The authors concluded that high variability of systolic blood pressure was associated with an increased risk of all‐cause dementia but not an increased incidence of cognitive decline on neuropsychological examinations. Their results also showed significant positive correlations with all‐cause dementia risk but not when Alzheimer's disease‐related dementia and vascular dementia were considered separately.

Parkinson's disease is also a very common cause of dementia that, together with Lewy body dementia (a progressive disease with prominent extrapyramidal movements) and multiple system atrophy (a progressive neurodegenerative disorder), constitute the so‐called synucleinopathies. These receive this name as abnormally misfolded α‐synuclein aggregates are a common finding in the peripheral and central nervous system of patients suffering from these conditions.^2^, ^3^

Besides the cognitive impairment, the aforementioned pathologies characteristically share the existence of autonomic disturbances that cause both orthostatic hypotension and supine or nighttime hypertension leading to increased blood pressure variability.^4^

This topic is of special interest for us, since it is recently becoming more and more recognized that blood pressure variability influences on vascular events and ischemic diseases even over or independently to mean blood pressure values.^5^, ^6^

After reading the review, we still have an unresolved question: Is blood pressure variability, due to its recognized capacity to cause vascular damage, the cause of dementia or, is dementia, which is sometimes associated with dysautonomia, the cause of the increased variability of blood pressure?

We understand that both hypotheses could be true, but we do not understand how, according to the meta‐analysis results, blood pressure variability is related to all‐cause dementia but not individually with that of vascular origin or with other neurological causes of dementia, while considered separately. We would like to know the opinion about it from the authors.
